# The Enzymatic Doped/Undoped Poly-Silicon Nanowire Sensor for Glucose Concentration Measurement

**DOI:** 10.3390/s23063166

**Published:** 2023-03-16

**Authors:** Cheng-Chih Hsu, Wen-Kai Ho, Chyan-Chyi Wu, Ching-Liang Dai

**Affiliations:** 1 Department of Electro-Optical Engineering, National United University, No. 2 Lienda, Miaoli 36063, Taiwan; 2 Department of Electrical Engineering, Yuan Ze University, 135, Yuan-Tung Road, Chung-Li 32003, Taiwan; 3 Department of Mechanical and Electromechanical Engineering, Tamkang University, New Taipei 25137, Taiwan; 4 Department of Mechanical Engineering, National Chung Hsing University, Taichung 402, Taiwan

**Keywords:** nanowire sensor, glucose concentration, n-type doping

## Abstract

In this work, enzymatic doped/undoped poly-silicon nanowire sensors with different lengths were fabricated using a top-down technique to measure glucose concentration. The sensitivity and resolution of these sensors correlate well with the dopant property and length of nanowire. Experimental results indicate that the resolution is proportional to the nanowire length and dopant concentration. However, the sensitivity is inversely proportional to the nanowire length. The optimum resolution can be better than 0.02 mg/dL for a doped type sensor with length of 3.5 μm. Furthermore, the proposed sensor was demonstrated for 30 applications with similar current-time response and showed good repeatability.

## 1. Introduction

In 2020, the U.S. Centers for Disease Control and Prevention (CDC) reported the latest scientific data on diabetes in the United States, indicating that about 10% (29.1 million) of the U.S. population suffers from diabetes. In 2012, diabetes-related treatment costs in the United States were estimated to be approximately $245 billion [1]. The International Diabetes Federation reported that 382 million people were living with diabetes in 2013 and this number is expected to increase to 592 million by 2035 [2]. In low- and middle-income countries, diabetes continues to be a growing health burden and will increase significantly in the coming decades. A large number of clinical studies have shown that controlling lower blood glucose levels can reduce the risk factors of cardiovascular diseases. Self-monitoring of blood glucose levels can be effectively performed using a commercial glucometer. According to the guideline suggested by the U.S. National Institute of Health (NIH), patients with type 2 diabetes typically self-test before meals, after meals, and at bedtime [1,3]. Therefore, measuring glucose concentration with a glucometer is critical to reducing the costs associated with diabetes.

There are various methods [4,5,6,7,8,9,10,11,12,13,14,15,16,17,18,19,20] to measure blood glucose concentration, which can be divided into non-invasive [4,5,6,7,8,9] and invasive [10,11,12,13,14,15,16,17,18,19,20]. Since the optical method [4,5,6,7,8,9] can realize the non-invasive measurement of blood glucose concentration, it has become a focus for technological development in recent decades. Although non-invasive blood glucose monitoring technology has the advantage of painless monitoring of blood glucose concentration, individual patient differences (including race, skin color, skin composition thickness, and complex blood components) will lead to various errors in measurement results and reduce the reliability of clinical diagnosis. Therefore, currently available methods for clinical monitoring of blood glucose levels are based on the invasive (in vitro) methods. To avoid the influence of complex chemicals from blood, these methods applied a specific enzyme to catalyze glucose into a unique resultant, which induced changes in the physical properties of the testing sample (such as refractive index (RI) [7], light intensity and polarization state [8], wavelength shift [9], or electric current [10,11,12,13,14,15,16,17,18,19,20]). Thus, these methods provide less individual variability and high reliability. Many studies [10,11,12,13,14,15,16,17,18,19,20] pointed out the possibility of measuring glucose concentration using nanowire-structured sensors. Nanowire sensors work on the principle that the charges around the structure will create a surface potential on the nanowire surface, which translates into a change in drain–source current [11,12]. Chen et al. [13] reviewed the current literature on Si nanowire field effect transistors (FETs) for biosensing and highlighted some advantages of enzyme-modified FETs for real-time quantitative analysis. Rahman et al. [14] comprehensively reviewed nanostructured metal oxide glucose biosensors and highlighted the advantages and drawbacks of enzymatic and non-enzymatic glucose sensors. The enzymatic glucose sensor exhibits high selectivity under appropriate pH conditions. Despite the better pH immunity of non-enzymatic sensors, the lower selectivity and rapid inactivation of such sensors remains a priority. Shao et al. [15] developed silicon nanowires with boron modification for glucose concentration measurement. On the surface of the boron modified silicon nanowire, a Donnan potential occurred and varied with the glucose concentration. The resistance of the nanowire sensor was measured to obtain the glucose concentration. The results showed good sensitivity and around 172 nAmM^−1^. Shervedani et al. [16] fabricated an enzyme-free glucose sensor using 3D spiny nickel nanowire on a copper substrate. The novel sensor structure provided good enhancement of electrocatalytic behavior for glucose oxidation. The sensitivity of their method could be better than 4000 μAcm^−2^mM^−2^ and the detection limit could be down to 0.1 μM. Li et al. [17] proposed a nonenzymatic glucose sensor with Pt-Pd nanowire arrays. Based on the electrochemical active surface area caused by the effective channel of the hydrogen adsorption/desorption, the sensors provided the sensitivity of 41.5 μAcm^−2^mM^−2^. Horng et al. [18] demonstrated an enzymatic glucose sensor that incorporated glucose oxidase (GOx) and direct-growth polaniline nanowires (PANI-NWs). The PANI-NWs enhanced redox-response resulted from the larger reaction surface area and lower resistance. The sensitivity could be better than 2.5 mAcm^−2^mM^−2^ and the detection limit would be lower than 0.05 mM. Dawson et al. [19] employed a redox mediator (FcCOOH) into an enzymatic gold nanowire glucose sensor. As a result of eliminating the oxygen consumption and interference effects of the mediator, the novel sensor amplified the electrochemical signal and therefore the detection limit could be down to 3 μM. Previous study [20] proposed un-doping multiple silicon nanowire sensor with enzymatic modification for measuring glucose concentration. The sensitivity of the nanowire sensor is inversely proportional to the length, and the sensor can take 10 consecutive measurements with similar results.

In this work, the top-down method and the technique of immobilizing glucose oxidase on nanowires were used to prepare doped/undoped poly-silicon nanowire sensors to measure glucose concentration. Using re-oxidation and oxide stripping procedures, the nanowire width was trimmed to a size of 130 nm with two different nanowire lengths (3.5 μm and 5.3 μm). The n-type (phosphorus) dopant was selected and the concentration was controlled at 5 × 10^18^ cm^−3^. Experimental results show that the sensitivity is inversely proportional to the length of the nanowire. The highest sensitivity of 0.008 µA/(mg/dL) can be obtained when the nanowire length is 3.5 μm. Theoretical and actual resolutions of the proposed sensor are 0.013 mg/dL and 1.75 mg/dL, respectively. Reliability is demonstrated by calculating the relative standard deviation (RSD), and the average RSD of 30 replicates for each glucose concentration can be better than 10%. Furthermore, the proposed sensor can be reused 30 times at an acceptable performance level. 

## 2. Fabrication Procedure for the Enzymatic Poly-Silicon Nanowire Glucose Sensor

**Figure 1:**
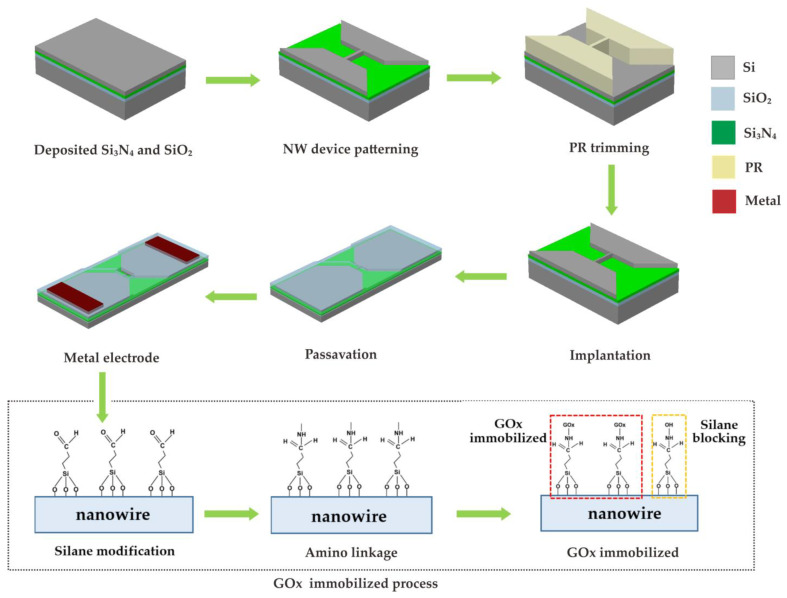
Schematic of the fabrication procedures of NWGS.

Figure 1 shows the fabrication process for the proposed nanowire glucose sensor (NWGS) with nanowires of different length, and the details are described below. In this work, two lengths of doped/undoped nanowires were fabricated under the same fabrication conditions. The proposed nanowire sensor was fabricated using a top-down method [13,16] and the silicon nanowire features are completed using nanopatterned I-line lithography. A 35 nm thick oxide layer was grown using thermal oxidation at 900 °C, followed by 30 nm thick Si_3_N_4_ deposited using low pressure chemical vapor deposition method (LPCVD) at 780 °C. Next, a layer about 2 nm thick was grown on the silicon nitride as the bottom dielectric layer using thermal oxidation at 900 °C. A 60 nm thick poly-silicon layer was deposited for the channel using LPCVD at 550 °C. Thereafter, I-line lithography and the photoresistor trimming process were performed together, followed by Si etching. Therefore, a source/drain pad region was formed on the bottom dielectric layer with n-type dopant (concentration of 5 × 10^18^ cm^−3^) by ion implantation. To adjust the dimensions of poly-silicon nanowire, a 30 nm thick thermal oxidation was performed at 900 °C followed by removal of the oxide. Finally, a poly-silicon nanowire width of about 130 nm could be reduced after the re-oxidation and oxide lift-off process. According to the previous study, short nanowire length exhibited high resolution [21]. Therefore, the length of the nanowires was controlled to around 3 μm to 5 μm in this study. The n-type (phosphorus) dopant was implanted and concentration was controlled at 5 × 10^18^ cm^−3^. 

**Figure 2:**
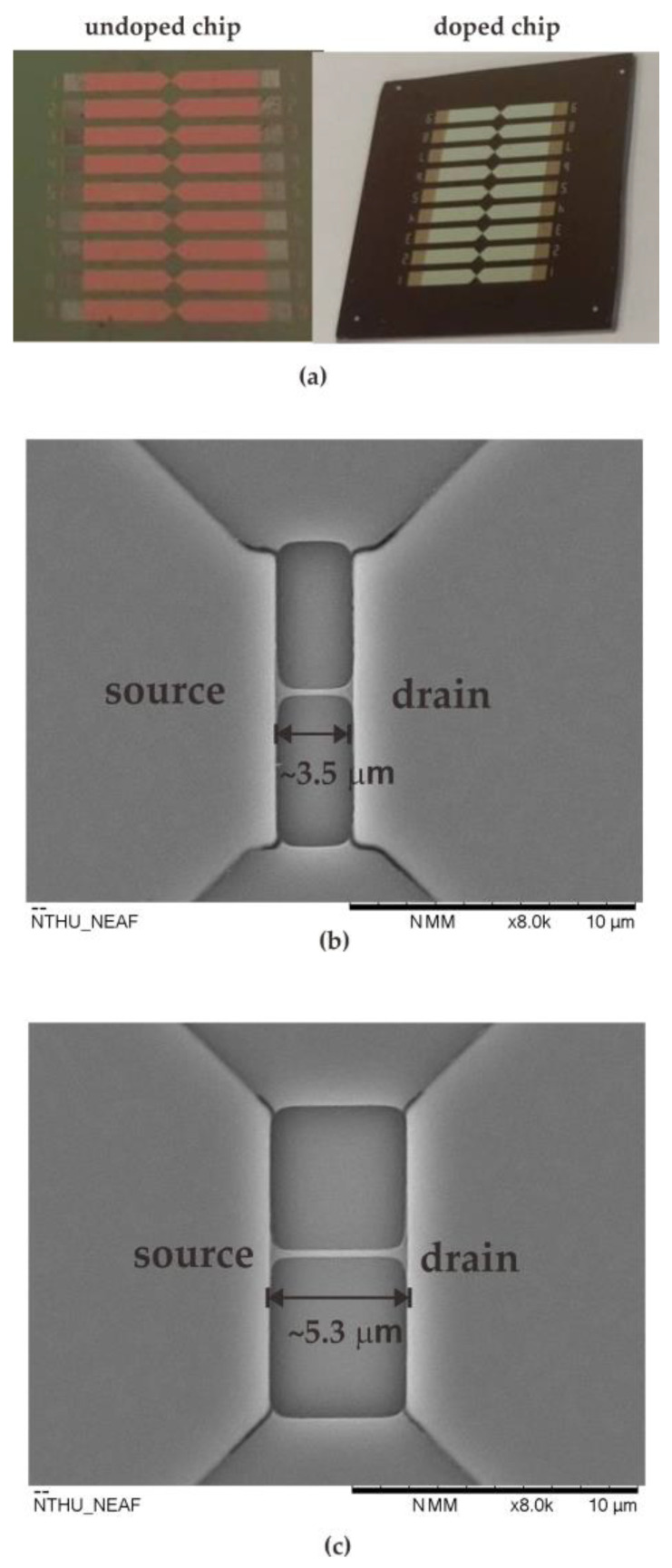
Photographs and top-view SEM images of NWGS. (**a**) photographs of the NWGS; (**b**) top-view SEM image of doped/undoped nanowire sensor with length of 3.5 μm; (**c**) top-view SEM image of doped/undoped nanowire sensor with length of 5.3 μm.

After fabricating the nanowire devices, the devices were covered with a 10% (*v*/*v*) ethanol solution of 3-aminopropyltriethoxysilane (APTES) for 15 min at room temperature. The solution was then removed and baked at 100 °C for 45 min to modify the nanowire’s surface. To covalently immobilize GOx on SiO_2_, 0.01% suberic acid bis(3-sulfo-N-hydroxysuccinimide ester) sodium salt (BS3) was mixed in 10 mM phosphate buffered saline (PBS) solution, and the mixture covered the nanowire surface for 30 min at room temperature. The modified surface was covered with a 150 µg/mL GOx solution at pH 7 for 1 h. Unreacted aldehyde groups were quenched by immersion in 15 mM Tris buffer solution for 15 min at room temperature [20]. Figure 2a shows the photographs of the NWGS with doped/undoped process and the top-view SEM images of NWGS with various lengths are shown in Figure 2b,c. It is clear that the length of the nanowire is well controlled at around 3 μm and 5 μm.

## 3. Theoretical Simulation

**Figure 3:**
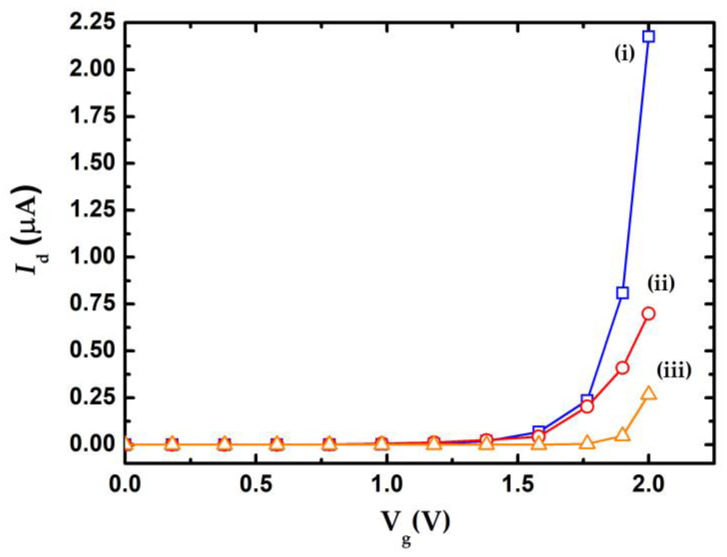
Simulation results of *I*_d_-V_g_ curve for various dopant concentrations of the poly-silicon nanowire.

The electrical behavior of nanowire can be described using the *I*_d_-V_g_ and *I*_d_-V_d_ curves. Different physical dimensions and dopant properties of the nanowires lead to different *I*_d_-V_g_ curve behaviors, indicating that the appropriate geometry and dopant concentration of the nanowires exhibited high sensitivity for biosensing purposes. Eflstrom et al. [12] indicated that the device sensitivity increased with decreased nanowire width. Previous work [20] reported that the threshold voltage and resolution of nanowires increased with increasing nanowire length. Cui et al. [22] showed that the resistivity of n-type nanowires exhibited more than 4 orders of magnitude smaller than the undoped nanowires. They also pointed out the n-type nanowire exhibited lower Fermi level when operated at the V_g_ > 0 condition, in which electrons will accumulate in nanowire and enhanced conductivity. These enhancements might be increasing the sensitivity of nanowire. This work simulates the *I*_d_-V_g_ curve of the nanowire under various dopant concentrations using commercial software (Synopsys, TCAD) by setting of the nanowire length and width to be 3 μm and 150 nm, respectively. The drain voltage was 0.5 V. Figure 3a indicates that the *I*_d_-V_g_ curve of various dopant concentrations of the nanowire, in which the curves (i) to (iii) corresponded to dopant concentration 1 × 10^18^ cm^−3^, 1 × 10^17^ cm^−3^, and undoped, respectively. Higher dopant concentration of the nanowire implies a larger variation of drain current.

**Figure 4:**
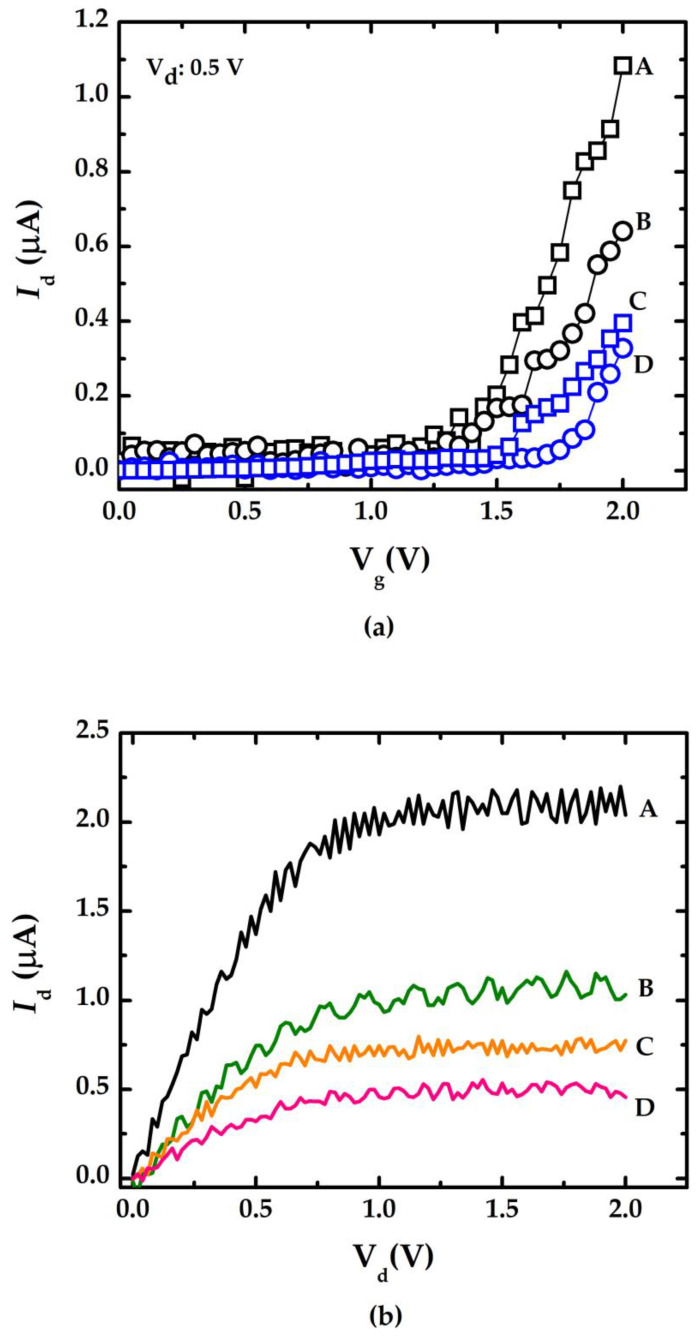
Electrical characteristic curves of NWGS with lengths of 3.5 μm and 5.3 μm. (**a**) *I*_d_-V_g_ measurement for doped/undoped poly-silicon nanowire with different nanowire length; (**b**) *I*_d_-V_d_ measurement for doped/undoped poly-silicon nanowire with different nanowire length.

Figure 4a summarizes the *I*_d_-V_g_ curve measurement results of doped/undoped NWGS with the lengths of 3.5 μm (for A and C) and 5.3 μm (for B and D), where labels A and B indicate the doped type NWGS, and labels C and D represent the undoped type NWGS. The dopant concentration of NWGS was controlled at 5 × 10^18^ cm^−3^. It is clear that the variation of *I*_d_ increases with increasing V_g_, and the threshold voltage V_th_ can be determined by the intercept of the V_g_-axis in the *I*_d_-V_g_ curve [11,12]. The threshold voltages (V_th_) of those NWGS are approximately 1.3 V (curve A), 1.25 V (curve B), 1.43 V (curve C), and 1.67 V (curve D), respectively. The results show that V_th_ decreases with the increase in nanowire dopant concentration. Figure 4b shows that the *I*_d_-V_d_ curves of the doped/undoped NWGS with the gate voltage controlled at 2 V. The results show the V_d_ should be within 0.75 V to maintain the device operation in a linear range. The results show that the doped NWGS with a shorter length has a more sensitive *I*_d_-V_d_ response, and its response level is about 2 times that of the doped NWGS with longer length and 6 times that of the undoped NWGS.

## 4. Experimental Results

**Figure 5:**
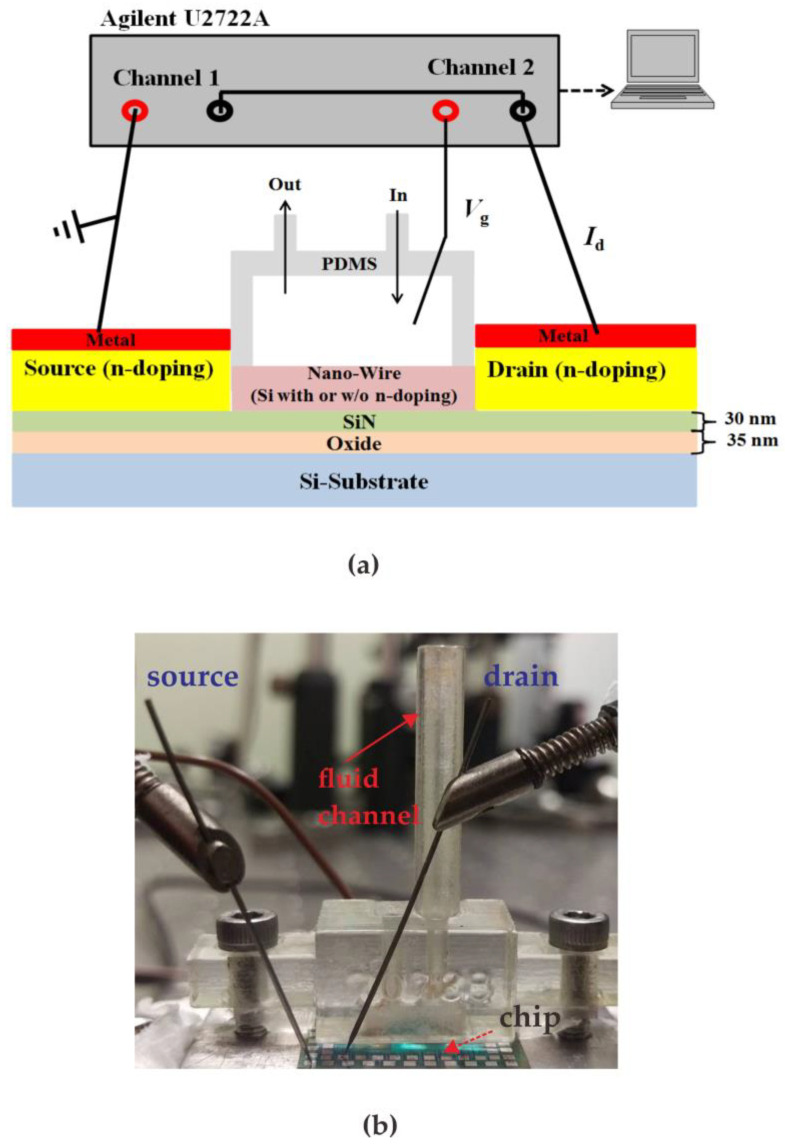
The measurement system of proposed method. (**a**) Diagram of the experimental setup; (**b**) real picture of the measurement system.

Figure 5a shows a measurement setup similar to Bergveld’s method [23] and Figure 5b shows an actual photo of the proposed system. Performance of the proposed poly-silicon nanowire sensor was demonstrated by measuring various glucose concentrations prepared by dissolving different weights of glucose in DI water. The test sample with a volume of 1 cc was injected from the injection port of the microfluidic channel and reacted with the NWGS for 10 s. Next, the reacted liquid was sucked out through the microfluidic channel’s out port while 10 cc of DI water was injected from the injection port for NWGS cleaning. The current meter (Agilent U2722A) has a resolution of 1 nA in the ±10 μA current range. In this study, the V_g_ and V_d_ were controlled at 2 V and 0.5 V, respectively. This study will verify the repeatability test and reliability verification of the NWGS, respectively.

**Figure 6:**
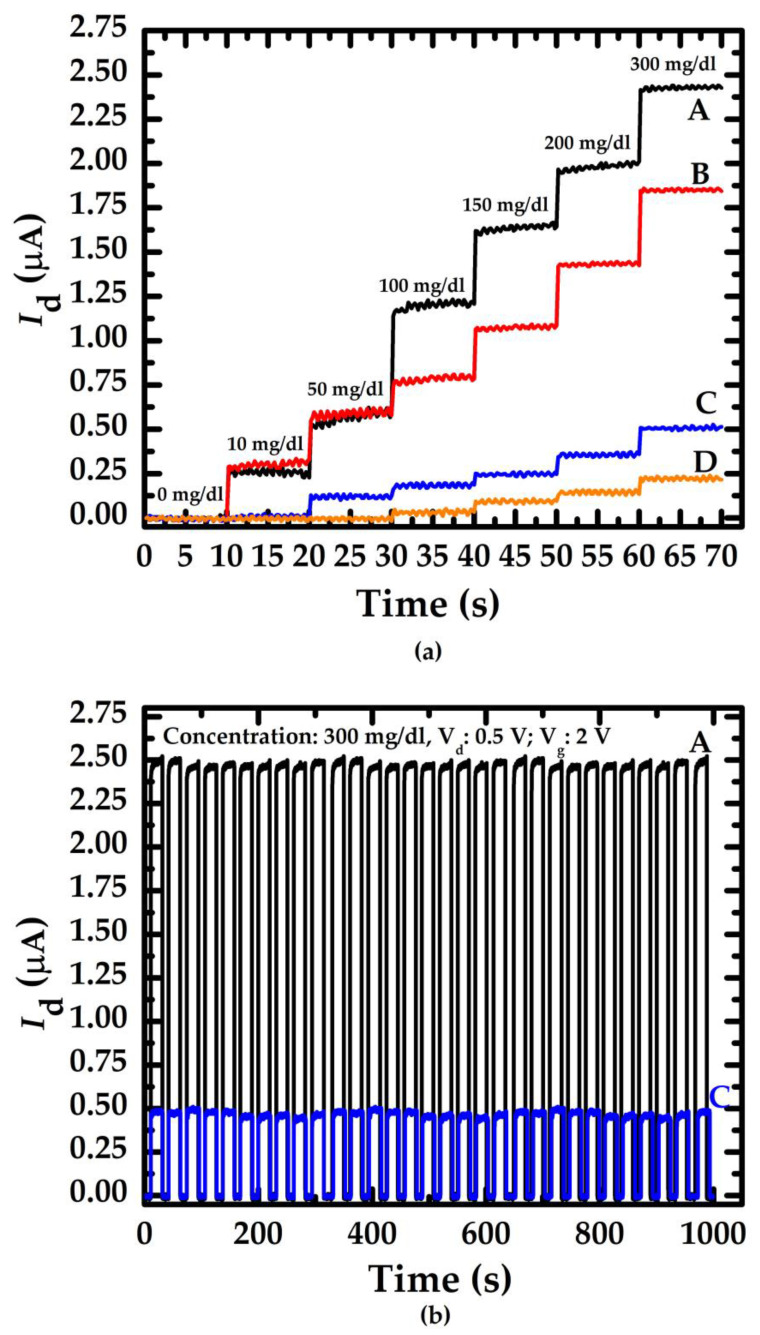
Current-time response curve of doped/undoped poly-Silicon nanowire with various nanowire length. (**a**) Relationship between the drain current and concentration of glucose; (**b**) Repeatability of the doped/undoped nanowire sensor with length of 3.5 μm.

To validate the NWGS repeatability test, glucose concentrations were varied from 10 mg/dL to 300 mg/dL and measured using the NWGS. Figure 6a,b shows the current-time response curve of the NWGS during the continuous additions of glucose solution. Figure 6a indicates that the drain current increases with an increasing concentration of glucose. It is clear that the current-time response curves of the doped type NWGS increase significantly as the glucose solution concentration is lower than 10 mg/dL. On the contrary, undoped type NWGS have no obvious change in drain current until the glucose concentration is greater than 50 mg/dL. The reason might derive from the dopant nanowire sensor reducing the resistivity [22] of the nanowire and lead to the greater sensitivity of the current-time response of nanowire sensor.

The repeatability of the proposed sensor was verified by performing 30 consecutive measurements on a glucose concentration of 300 mg/dL, where sensors A and C were selected for comparison, as shown in Figure 6b. The average values of *I*_d_ of sensor A and C were 2.41 μA and 0.45 μA, respectively. Moreover, the standard deviation (SD) of *I*_d_ corresponding to each sensor was 0.06 and 0.14, respectively. Clearly, Figure 6b shows that the current-time response curves exhibit high similarity in 30 consecutive applications of each sensor.

**Figure 7:**
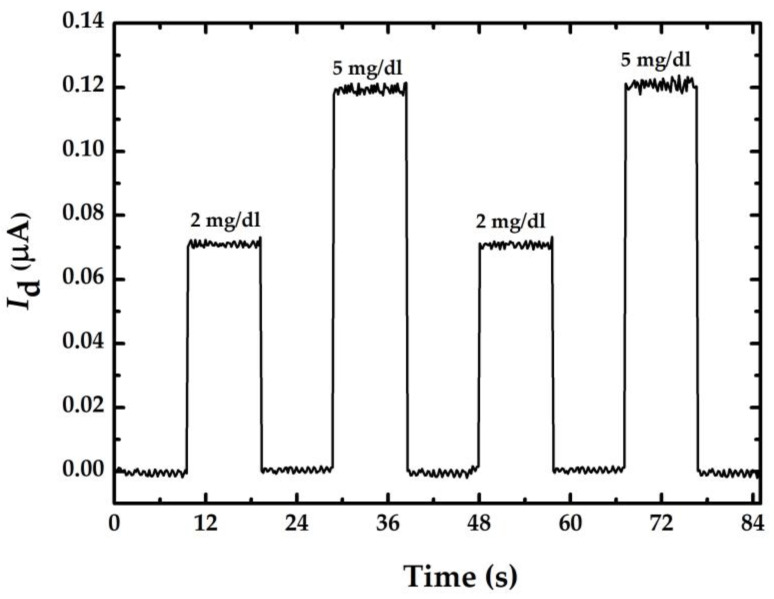
Current-time response curve for smallest glucose concentration measurement using a doped nanowire sensor with a length of 3.5 μm.

Figure 7 shows the smallest glucose concentration measurement with sensor A. The results show that the difference of *I*_d_ between 5 mg/dL and 2 mg/dL is about 50 nA. Considering the resolution of the current meter (0.1 nA), sensor A should theoretically be able to measure a change of 1 mg/dL. Therefore, the proposed sensor with short nanowire length and n-type doping has a high possibility of measuring the glucose concentration below 1 mg/dL.

**Figure 8:**
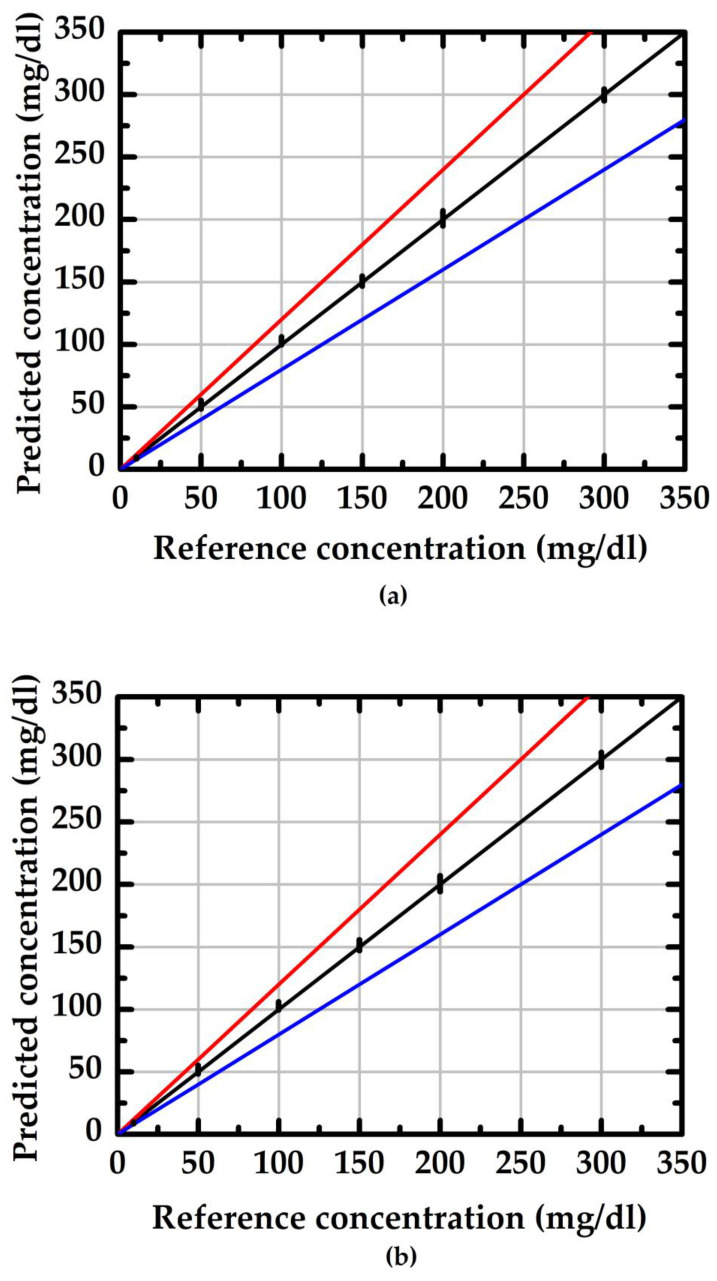
The reliability evaluation of the proposed method with different length of the doped type NWGS. (**a**) nanowire lengths of 3.5 μm; (**b**) nanowire lengths of 5.3 μm.

**Figure 9:**
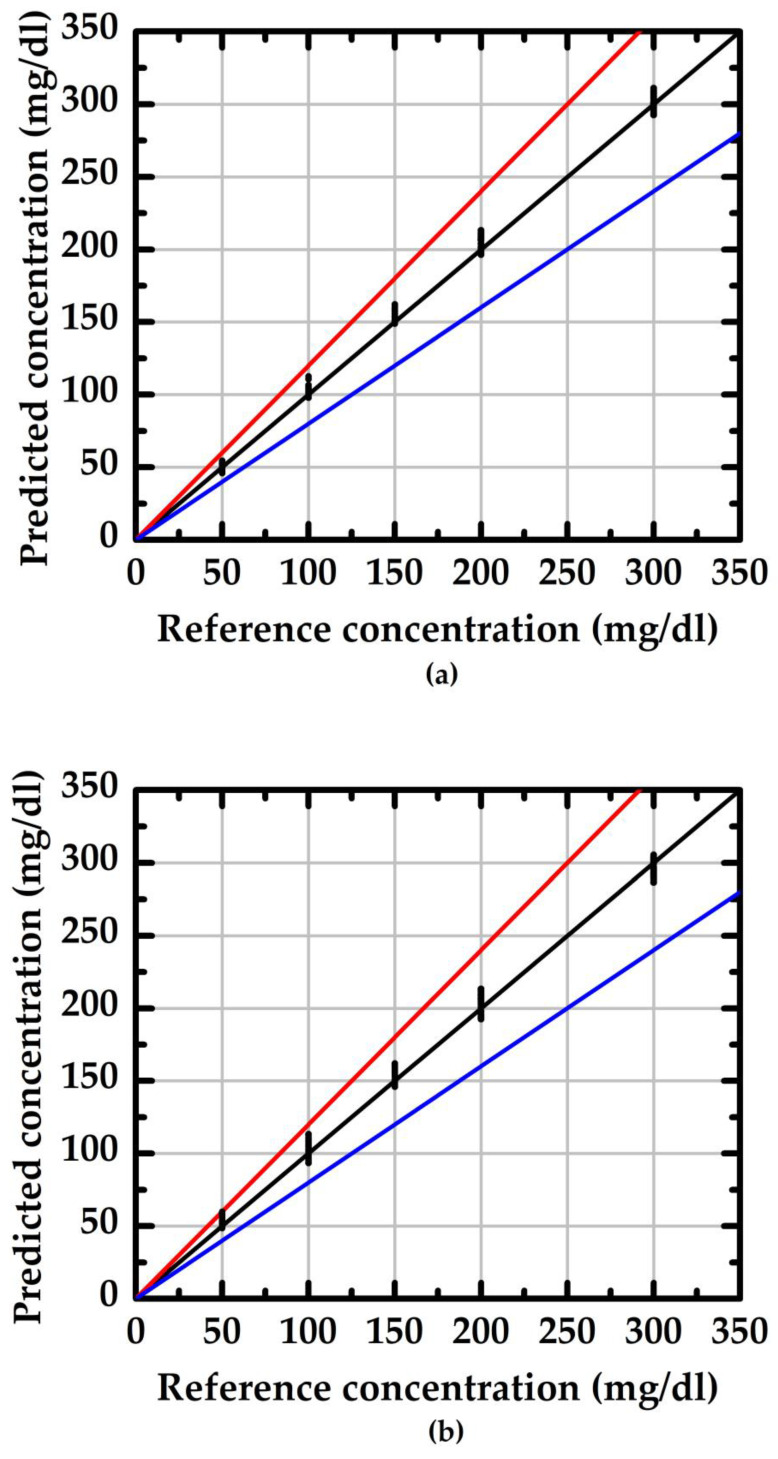
The reliability evaluation of the proposed method with different length of the undoped type NWGS. (**a**) nanowire lengths of 3.5 μm; (**b**) nanowire lengths of 5.3 μm.

The reliability evaluation was performed by calculating the relative standard deviation (RSD) of 30 replicate experiments for each glucose concentration in accordance with Clarke’s method [24]. The RSD can be expressed as

(1) $$\mathrm{RSD}=\frac{100\times \mathrm{SD}}{\bar{x}}$$

where SD is standard deviation and $\bar{x}$ is the average value of the measured data. Using doped/undoped NWGS and a traceable refractometer (reference method, model: excellence R4, Mettler) to conduct 30 groups of measurements, the evaluation results are shown in Figure 8 and Figure 9. The values in the region between the red and blue lines indicate the measurement result within 20% of the reference concentration. The average RSD of the doped type NWGS with lengths of 3.5 μm and 5.3 μm are 4% and 5%, respectively. The average RSDs for undoped NWGS are 9% and 10%, respectively. These results demonstrate good reliability of the proposed method for both types of NWGS and satisfied the minimum requirement (±15%) for point-of-care devices provided by the Food and Drug Administration (FDA) [25].

## 5. Discussion

**Figure 10:**
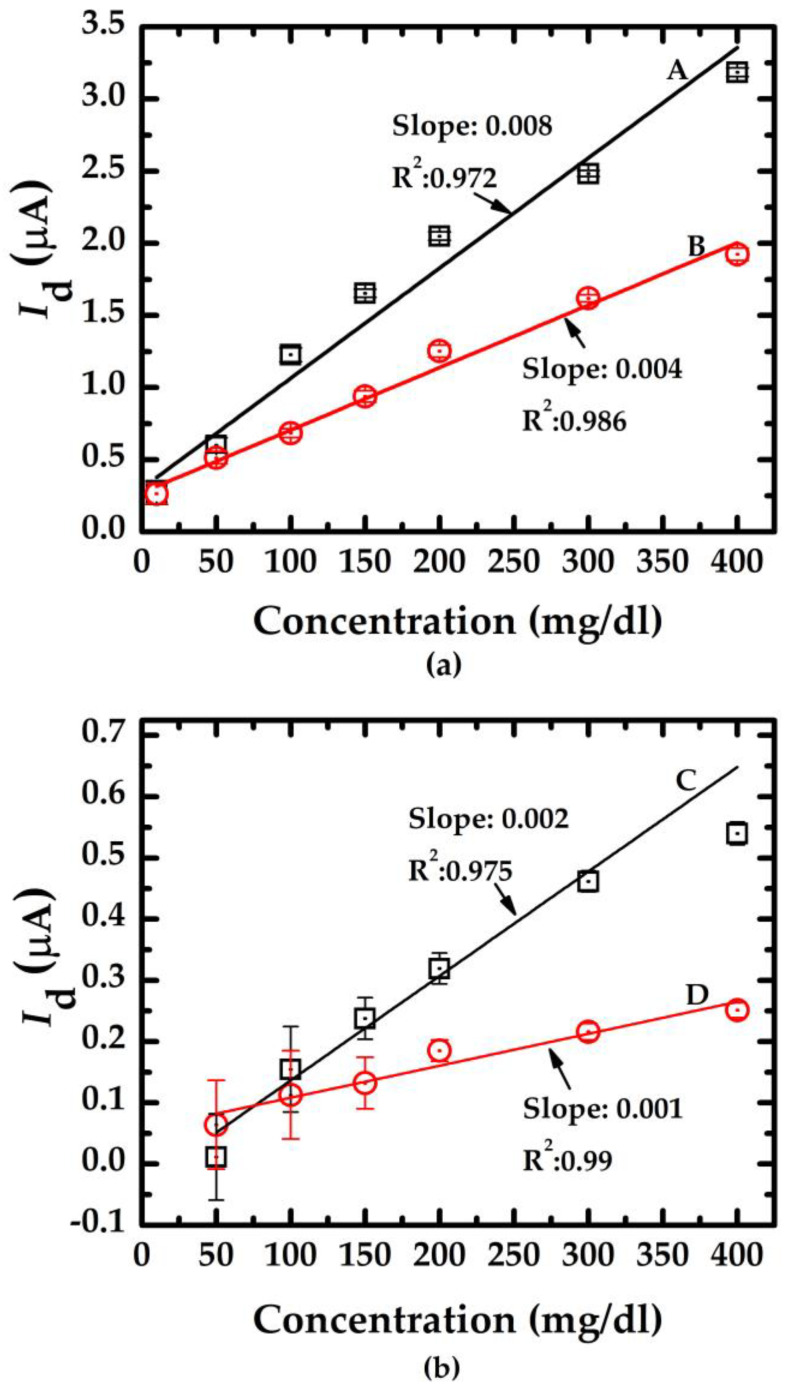
The calibration curves of the salt concentration measurement of the doped/undoped poly-silicon nanowire sensors. (**a**) doped type NWGS with lengths of 3.5 μm (A) and 5.3 μm (B); (**b**) undoped type NWGS with lengths of 3.5 μm (C) and 5.3 μm (D).

Figure 10 indicates the resulting calibration curve measured using the doped/undoped nanowire sensor. The symbols ☐, ◯, and I represent the average value of 10 measured data sets and the standard deviation of each concentration measured using the proposed sensor with doped/undoped nanowire sensors, respectively. Figure 10a shows the results of the measurements of doped nanowire sensors with nanowire of various lengths (labeled A and B with lengths of 3.5 μm and 5.3 μm, respectively). Figure 10b shows the results of the measurements of undoped nanowire sensors with nanowire of various lengths (labeled C and D with lengths of 3.5 μm and 5.3 μm, respectively). The slope of the calibration curve indicates the sensitivity (change in current per unit concentration change) of the proposed sensor and the fact that the nanowires with shorter lengths from the device with n-type dopant exhibited higher sensitivity. This finding correlates with the *I*_d_-V_g_ curves in Figure 4. The shaper slope indicates a higher sensitivity.

**Figure 11:**
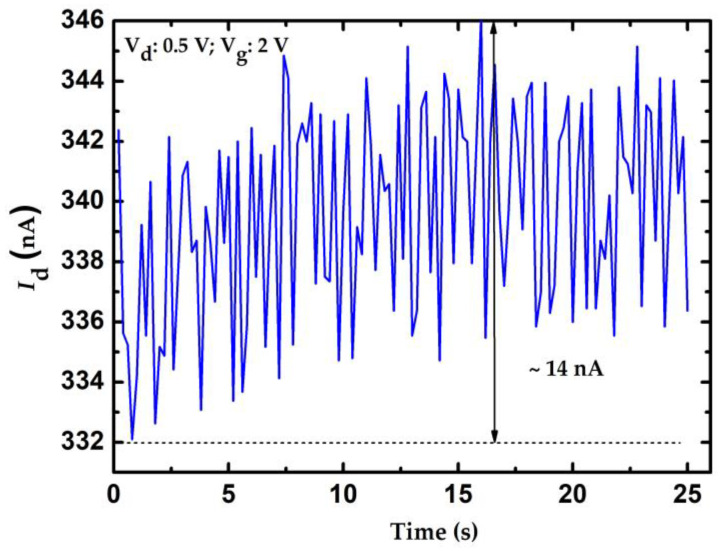
Current fluctuation measurement of the proposed system.

Resolution of the proposed method can be represented as [26]

(2) $$C_{res}=\frac{\Delta I}{S}$$

where *S* is the slope of the calibration curve; and ∆*I* and *C_res_* represent the resolution of the current measurement and glucose concentration, respectively. Resolution of the measured current depends on the current meter. The resolution of the current meter is 0.1 nA which can be considered as the theoretical resolution of the proposed method. There are many issues that can reduce the resolution of amperometric measurement, such as the temperature variation, conductivity fluctuations of the electrodes, and unwanted electrical noise from the measurement equipment. To evaluate the practical resolution of the measured current of the proposed system, the current fluctuation of the proposed system can be considered by applying a constant V_g_ to the DI water sample. In this work, the current fluctuation is approximately 14 nA within 25 s and the results are shown in Figure 11. According to Equation (2), the resolution of these sensors can be calculated and are shown in Table 1. Clearly, doped NWGS exhibited higher resolution than undoped NWGS, and the resolution of NWGS with shorter nanowire length was higher than that of NWGS with longer nanowire length. An n-type dopant sensor with a nanowire length of 3.5 μm can achieve the optimum resolution for the proposed sensor which is approximated of 1.75 mg/dL.

The threshold voltage (V_th_) of nanowire device depends on the doping concentration, doping types, and the geometric structure of nanowire, such as length, width, and thickness. For nanowire structures with different combinations of length, width, and thickness, the V_th_ variation characteristics of the above parameters will also be different. Fong [27] pointed out that when the length of n-type nanowires was between 1–3 μm and drain voltage was controlled at 2 V, the change in V_th_ was proportional to the length of the nanowires. However, when drain voltage was 1 V, the change in V_th_ has no obvious relationship with the length of the nanowire. In this study, when n-type nanowires operate at V_d_ of 0.5 V, V_th_ decreases as the nanowire length increases. Under the same operation, the V_th_ of the undoped nanowires shows the opposite behavior. Furthermore, at the same length of nanowires operating at V_d_ of 0.5 V, V_th_ increases with increasing doping concentration.

The sensitivity of the nanowire sensor will depend on the doping concentration of nanowire and will also depend on the evaluation method. Nair et al. [28] evaluated the sensitivity using relative change in conductance at low drain bias and they concluded that sensitivity was inverse to the doping concentration. Li et al. [29] followed the concept and evaluated using normalized current method at V_d_ of 0.01 V. In contrast, this study directly measured the drain current at various concentrations and sensitivity was defined as the slope of the calibration curve of drain current versus concentration curve. The V_d_ is 20 times higher than for the Li [29] method and may induce unequal amounts of depleted/accumulated charge or molecule conjugation on surfaces with different surface densities. Therefore, that may be a possible reason why the sensitivity differs from other methods.

**Figure 12:**
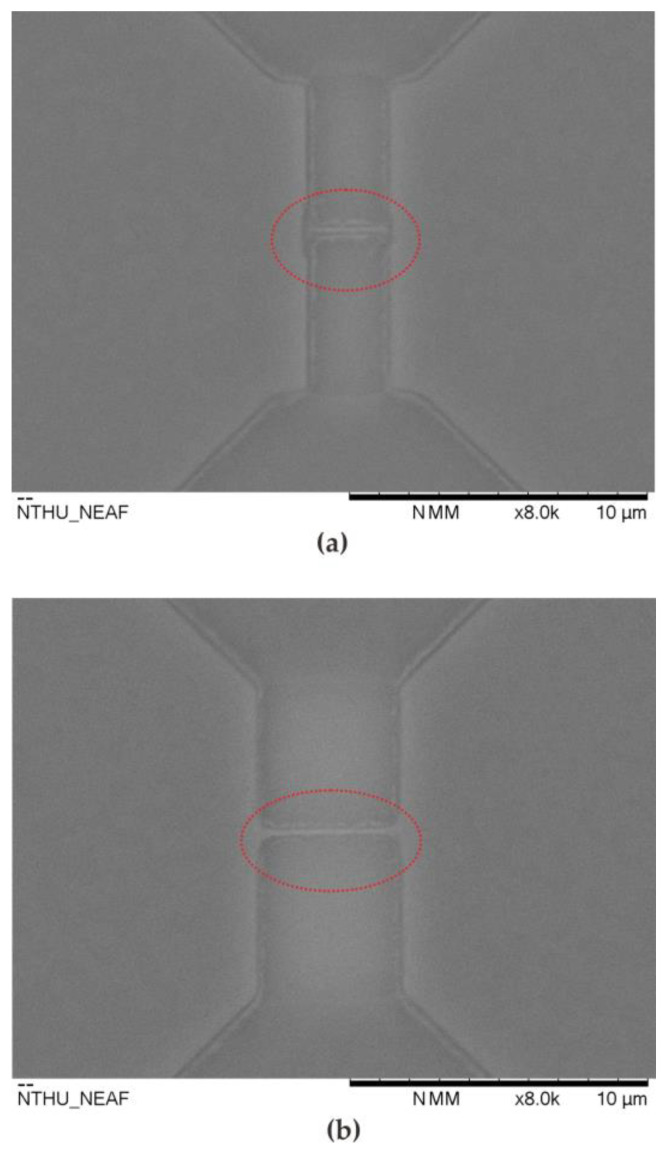
Top-view SEM images of the proposed sensor after 40 applications. (**a**) doped type nanowire sensors with lengths of 3.5 μm; (**b**) doped type nanowire sensors with lengths of 5.3 μm.

Figure 12 shows the SEM image of the top view of the sensor after 40 applications. Because the enzyme sensing layer catalyzes glucose and converts it to gluconic acid and hydrogen peroxide even after each reaction, the elements are cleaned with DI water. Improper cleaning methods still cannot ensure that the residual gluconic acid is completely removed. Therefore, gluconic acid is taken up by the device and deposited around the source, drain, and nanowire regions (marked in a red circle). Compared with previous work [20], the multi-nanowire structure has better measurement sensitivity. However, under the same sensor cleaning conditions, the reuse characteristics are worse than those of single nanowire sensors. Figure 12 shows that the single nanowire structure absorbs less residual gluconic acid, which is significantly better than the multi-nanowire structure. To prevent the absorption of gluconic acid, the orientation of the microfluidic channel can be adjusted to align with the orientation of the nanowires. In addition, the injection system can be replaced by an auto-injector, which provides continuous injection of deionized water to clean the device.

Table 2 summarizes the performance comparison results between the proposed sensor and the related work cited in Section 1 (in vitro method). The proposed method yields a reusable glucose sensor with acceptable detection limits, fast response time, and wide measurement range. It is worth mentioning that the reusable NWGS will help reduce the manufacturing cost of the sensor, reduce the generation of medical waste, and have less impact on the environment. Therefore, it will become a green medical product.

## 6. Conclusions

This work demonstrates the feasibility of a doped/undoped poly-silicon nanowire sensor for measuring the concentration of glucose. Experimental results show that the resolution of the proposed nanowire sensor is strongly related to the length and dopant characteristics of the nanowire. The resolution increases with decreasing nanowire length and increasing dopant concentration. Moreover, the best resolution is approximately 1.75 mg/dL and the smallest concentration variation that can be determined is 2 mg/dL. This work further demonstrates the repeatability of the proposed sensor, indicating that the applications within 30 times can be sustained with an acceptable current-time response for doped/undoped nanowire sensors.

## Figures and Tables

**Table 1:** The resolution of the doped/undoped NWGS.

Item	*S* (μA/(mg/dL))	Theoretical		Practical	
		Δ*I* (nA)	*C_res_* (mg/dL)	Δ*I* (nA)	*C_res_* (mg/dL)
A	0.008	0.1	0.013	14	1.75
B	0.004		0.025		3.5
C	0.002		0.05		7
D	0.001		0.1		14

**Table 2:** Performance comparison of nanowire glucose sensor.

Ref.	Detection Limit	Linear Range	Response Time	Reusability	Sample Type	Enzyme Adopted
[15]	0.16 mM	0–10 mM	X	X	D-glucose	X
[16]	0.1 μM	3 μM–2 mM	X	X	D-glucose	X
[17]	1.31 mM	0–10 mM	>20 s	X	D-glucose/Rabbit blood serum	X
[18]	1 mM	0–8 mM	X	X	D-glucose	GOx
[19]	3 μM	10 μM–100 mM	X	X	D-glucose	GOx
[20]	1.23 mg/dL	10–300 mg/dL	<5 s	10 times	D-glucose	GOx
This work	1.75 mg/dL	0–400 mg/dL	<5 s	30 times	D-glucose	GOx

## Data Availability

Not applicable.
